# Rasd1, a small G protein with a big role in the hypothalamic response to neuronal activation

**DOI:** 10.1186/s13041-015-0182-2

**Published:** 2016-01-07

**Authors:** Michael P. Greenwood, Mingkwan Greenwood, Andre S. Mecawi, José Antunes-Rodrigues, Julian F. R. Paton, David Murphy

**Affiliations:** School of Clinical Sciences, University of Bristol, Bristol, BS1 3NY UK; School of Medicine of Ribeirão Preto, University of São Paulo, Ribeirão Preto, Brazil; Department of Physiology, University of Malaya, Kuala Lumpur, 50603 Malaysia; Department of Physiological Sciences, Biology Institute, Federal Rural University of Rio de Janeiro, Seropedica, Rio de Janeiro Brazil; School of Physiology and Pharmacology, University of Bristol, Bristol, BS8 1TD UK

## Abstract

**Background:**

*Rasd1* is a member of the *Ras* family of monomeric G proteins that was first identified as a dexamethasone inducible gene in the pituitary corticotroph cell line AtT20. Using microarrays we previously identified increased *Rasd1* mRNA expression in the rat supraoptic nucleus (SON) and paraventricular nucleus (PVN) of the hypothalamus in response to increased plasma osmolality provoked by fluid deprivation and salt loading. RASD1 has been shown to inhibit adenylyl cyclase activity in vitro resulting in the inhibition of the cAMP-PKA-CREB signaling pathway. Therefore, we tested the hypothesis that RASD1 may inhibit cAMP stimulated gene expression in the brain.

**Results:**

We show that *Rasd1* is expressed in vasopressin neurons of the PVN and SON, within which mRNA levels are induced by hyperosmotic cues. Dexamethasone treatment of AtT20 cells decreased forskolin stimulation of *c-Fos*, *Nr4a1* and phosphorylated CREB expression, effects that were mimicked by overexpression of *Rasd1*, and inhibited by knockdown of *Rasd1*. These effects were dependent upon isoprenylation, as both farnesyltransferase inhibitor FTI-277 and CAAX box deletion prevented *Rasd1* inhibition of cAMP-induced gene expression. Injection of lentiviral vector into rat SON expressing *Rasd1* diminished, whereas CAAX mutant increased, cAMP inducible genes in response to osmotic stress.

**Conclusions:**

We have identified two mechanisms of *Rasd1* induction in the hypothalamus, one by elevated glucocorticoids in response to stress, and one in response to increased plasma osmolality resulting from osmotic stress. We propose that the abundance of RASD1 in vasopressin expressing neurons, based on its inhibitory actions on CREB phosphorylation, is an important mechanism for controlling the transcriptional responses to stressors in both the PVN and SON. These effects likely occur through modulation of cAMP-PKA-CREB signaling pathway in the brain.

## Background

The hypothalamo-neurohypophyseal system (HNS) is the source of the neuropeptide hormone arginine vasopressin (AVP). AVP is synthesised in magnocellular neurons (MCN) of the supraoptic nucleus (SON) and paraventricular nucleus (PVN) and is transported anterogradely to terminals in the posterior pituitary gland. A rise in plasma osmolality increases secretion of AVP into the blood stream where it promotes water reabsorption at the kidney [1].

While the SON contains a homogenous population of MCN, the PVN is divided into MCN and parvocelluar neurons (PCNs). The PCNs form part of the hypothalamo-pituitary-adrenal (HPA) axis that mediates the stress response. In response to restraint stress, AVP and corticotropin releasing hormone (CRH) are released from the PCN axon terminals in the median eminence into the portal vasculature [2–4] that supplies the anterior pituitary to stimulate the release of adrenocorticotropin hormone [5, 6], and, subsequently, glucocorticoids from the adrenal cortex. These secretory responses are accompanied by transcriptional increases in *Avp* and *Crh* in PCN by stress [7–9] and *Avp* in MCN of the hypothalamus by osmotic stress [10].

The signaling mechanisms governing transcriptional increases in *Crh* and *Avp* are believed to involve cAMP activation of the protein kinase A (PKA) pathway and the subsequent phosphorylation of cAMP response element binding protein (CREB) [11]. It is known that both hyperosmotic and restraint stress increase the abundance of phosphorylated CREB, a process that occurs within minutes of stimulation in MCN and PCNs [8, 9, 12, 13]. Stress induced transcriptional increases can be short-lived, particularly for *Crh* and *Avp* in PCNs, as the subsequent increase in circulating levels of glucocorticoid following stress, through its interactions with glucocorticoid receptors (GR) within these neurons [14], rapidly dampens this transcriptional response. This feedback by glucocorticoids has been reported to inhibit CREB phosphorylation in PCNs [12], through a proposed unknown intermediate intracellular signaling molecule regulating cAMP [7]. Less is known about inhibitory inputs controlling the transcriptional response to osmotic stress in MCN of the PVN and SON where phosphorylated CREB levels also increase [13]. MCN of the SON express GRs [15], and expression has been shown to increase during hypoosmotic stress [16], indicating that glucocorticoid negative feedback is a possible route for regulation, though studies suggest considerably lower levels of this receptor compared to PCNs of the PVN [14, 17]. Nonetheless, we reasoned that a glucocorticoid inducible gene might be important for regulating transcriptional feedback inhibition in both MCN and PCNs.

Our candidate was *Rasd1* (dexamethasone inducible Ras protein 1, Dexras1), a member of the Ras family of monomeric G proteins that was first identified as a dexamethasone (DEX) inducible gene in the pituitary corticotroph cell line AtT20 [18]. A putative glucocorticoid response element identified by Kemppainen and colleagues [19] in the 3’ flanking region of the human *Rasd1* gene was shown to confer rapid responsiveness to glucocorticoids by reporter assay. Indeed, the peripheral administration of DEX in rats and mice strongly and rapidly induces *Rasd1* expression in several tissue types, including the brain [18, 20, 21]. Another important feature of *Rasd1* is its role as an inhibitor of adenylyl cyclase (AC) activity [22–25]. An in vitro study by Graham et al. [22] showed that *Rasd1* inhibits GαS- and forskolin (FSK)-induced increases in cAMP levels through ligand independent activation of the Gαi and Gβγ arms of the Gi signaling pathway. This results in inhibition of the cAMP-PKA-CREB signaling pathway and so forms a possible feedback mechanism for regulation of MCN and PCNs. There is also in vitro evidence from reporter assays that *Rasd1* acts at cAMP response element (CRE) sites within target gene promoters, to directly inhibit transcription [26, 27]. Thus, *Rasd1* has the capacity to influence transcriptional events either indirectly, by inhibiting AC activity at the cell membrane, or directly, through modulation of activity at the level of the promoter.

Using microarrays we identified increased *Rasd1* expression in the rat and mouse SON in response to hyperosmotic stress (dehydration) [28, 29]. *Rasd1* mRNA expression has previously been described in the mouse PVN and SON but its function in these brain nuclei is not known [30, 31]. Only in the suprachiasmatic nucleus (SCN) of the brain has *Rasd1* expression been interrogated thoroughly [32]. *Rasd1* cycles with a circadian rhythm that oscillates in antiphase with many of the cAMP inducible mRNAs in the SCN [31] including *Avp*. Interestingly, in the SCNs of *Rasd1* knockout mice, Cheng et al. [26] reported higher cAMP levels alongside increased CREB phosphorylation compared to wild-type mice. We thus hypothesised that *Rasd1* may control the transcription of cAMP inducible genes, and ultimately their downstream targets, *Avp* and *Crh*, in the hypothalamus. Using in vitro and in vivo approaches we demonstrate that *Rasd1* negatively regulates the hypothalamic transcriptional response to stimulation by hyperosmotic stress in the rat.

## Results

### *Rasd1* mRNA expression in the rat hypothalamus in response to different stressors

**Fig. 1:**
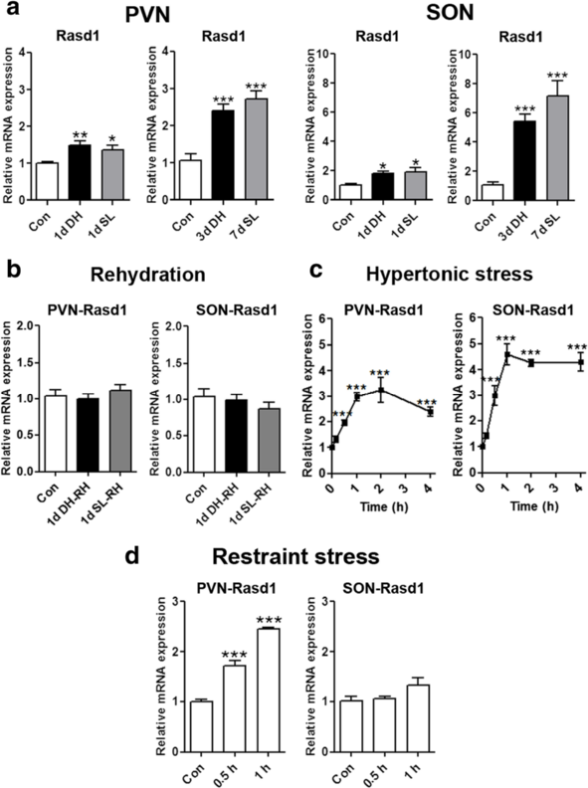
*Rasd1* mRNA expression in the rat PVN and SON in response of hyperosmotic stress (**a**–**c**). The expression of *Rasd1* was examined in the PVN and SON in chronic and acute hyperosmotic conditions, and following water repletion. Relative mRNA expression of *Rasd1* was investigated by qPCR in the PVN and SON of **a** control, DH (1 and 3 days), and SL (1 and 7 days) rats, **b** after water repletion for 1 day after 1 day DH and 1 day SL compared to controls, **c** after a single i.p injection of 1.5 ml/100 g body weight 1.5 M NaCl over a 4 h experimental period compared to controls. **d**, *Rasd1* mRNA expression in PVN and SON after 30 min and 1 h restraint stress compared to control. All data was analysed by One-way ANOVA. Values are means + SEM of *n* = 5−6 animals per group. *p < 0.05, **p < 0.01, ***p < 0.001. RH, rehydration; Con, control

We first investigated the time course of *Rasd1* mRNA expression in the PVN and SON of control, dehydrated (DH) and salt loaded (SL) rats using qPCR (Fig. 1a). *Rasd1* mRNA expression was significantly higher in the PVN and SON of 1 day (day) DH and 1 day SL rats compared with control rats. Increasing the duration of hypertonic stress to 3 days DH and 7 days SL further increased the magnitude of this response in both PVN and the SON. When drinking water was returned after 1 day DH and 1 day SL, *Rasd1* mRNA abundance was seen to quickly return to control levels (Fig. 1b).

We then examined the acute response of *Rasd1* transcripts to a single intraperitoneal (i.p) injection of hypertonic saline (HS) (Fig. 1c). The abundance of the *Rasd1* mRNA increased rapidly after injection in both PVN and SON, and levels progressively increased until peaking at around 60 min, with levels remaining steadily elevated thereafter. As *Rasd1* is known to be strongly induced by glucocorticoids [18], we also examined expression in PVN and SON resulting from stress induced by 30 min and 1 h restraint (Fig. 1d). An increase of *Rasd1* mRNA was observed in PVN but interestingly not the SON.

### Expression of RASD1 protein in the rat HNS

**Fig. 2:**
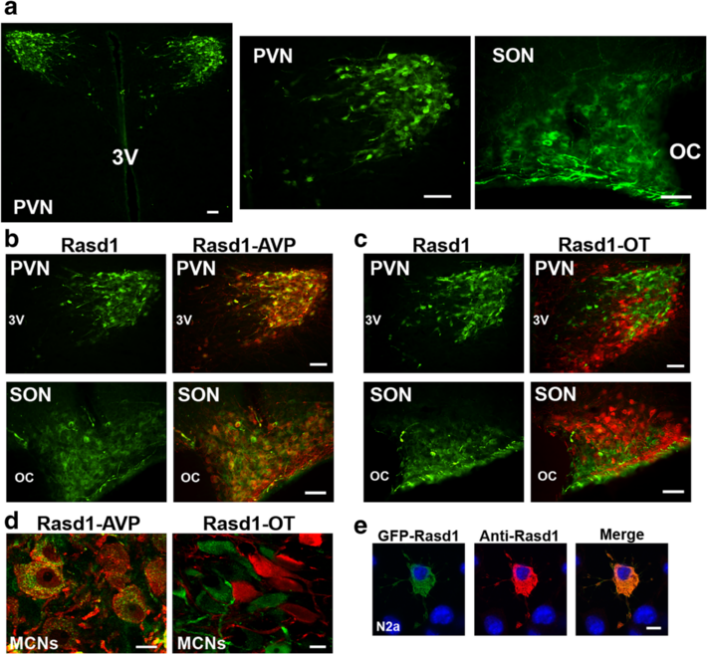
Immunofluorescent localisation of RASD1 in the hypothalamus of the euhydrated rat. **a**, immunofluorescent localisation of RASD1 in MCN of the PVN and SON. **b**–**c** immunofluorescent colocalisation of RASD1 (*green*) with **b** AVP (*red*) and **c** OT (*red*) in MCN in the PVN and SON. **d** Higher magnification images showed the presence of *Rasd1* in AVP, but not OT, positive cells. **e**, eGFP and *Rasd1* fluorescence in N2a cells transfected with eGFP-*Rasd1* fusion construct. Dapi staining indicates the nucleus. Scale bars **a**–**c** = 100 μm, **d**–**e** = 10 μm. OC, optic chiasm; 3 V, third ventricle

Immunofluorescent localisation studies were performed to determine the cell populations expressing RASD1 in the PVN and SON of control rats (Fig. 2). RASD1 was expressed MCN of the PVN and SON (Fig. 2a). In the PVN, RASD1 staining was predominantly found in the lateral magnocellular part with fewer RASD1 positive neurones in parvocellular regions. The MCN of the SON and PVN can be divided into two populations, expressing either AVP, or the closely related neuropeptide hormone oxytocin (OT), with only a small percentage (2–3%) of MCN express high, equivalent levels of both peptides [33]. Therefore, to examine the identity of neurons expressing RASD1 in the PVN and SON, co-immunostaining of RASD1 with AVP neurophysin-II or OT neurophysin-I was performed in euhydrated control rats (Fig. 2b–c). RASD1 expression was observed in AVP positive (Fig. 2b), but not OT expressing MCN of the PVN and SON (Fig. 2c). High magnification images using confocal microscopy are shown in Fig. 2d. Therefore, one can assign the potential functions of *Rasd1* principally to vasopressinergic neurones in these two brain nuclei following hyperosmotic stress. The specificity of the RASD1 antibody was confirmed in N2a cells transfected with GFP-*Rasd1* fusion construct (Fig. 2e).

### Effect of osmotic and restraint stress on RASD1 protein distribution

**Fig. 3:**
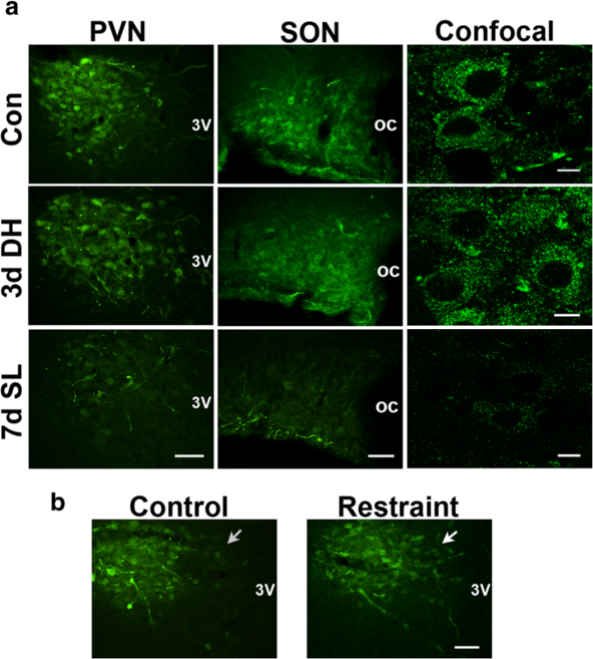
The effect of osmotic and restraint stress on RASD1 expression. **a**, immunofluorescent staining for RASD1 in the PVN and SON of control, 3 days DH and 7 days SL rat. High magnification confocal images are shown. **b**, immunofluorescent staining of RASD1 in the rat PVN in response 1 h restraint stress with 3 h recovery. Arrows indicate the dorsomedial parvocellular portion of PVN. Scale bars **a**–**b** = 100 μm, confocal images = 10 μm. OC, optic chiasm; 3 V, third ventricle; Con, control

Immunofluorescence staining was performed to examine the expression of RASD1 after exposure to either osmotic or restraint stress (Fig. 3). The intensity of RASD1 staining in neuronal cell bodies of the PVN and SON appeared similar in DH but lower in SL compared to control rats (Fig. 3a). We then looked at the distribution of RASD1 staining in the PVN after restraint stress (Fig. 3b). The degree of RASD1 staining appeared higher in the dorsomedial parvocellular portions in response to restraint stress (Fig. 3b).

### Activation of *Rasd1* transcription by glucocorticoids in the PVN and SON

**Fig. 4:**
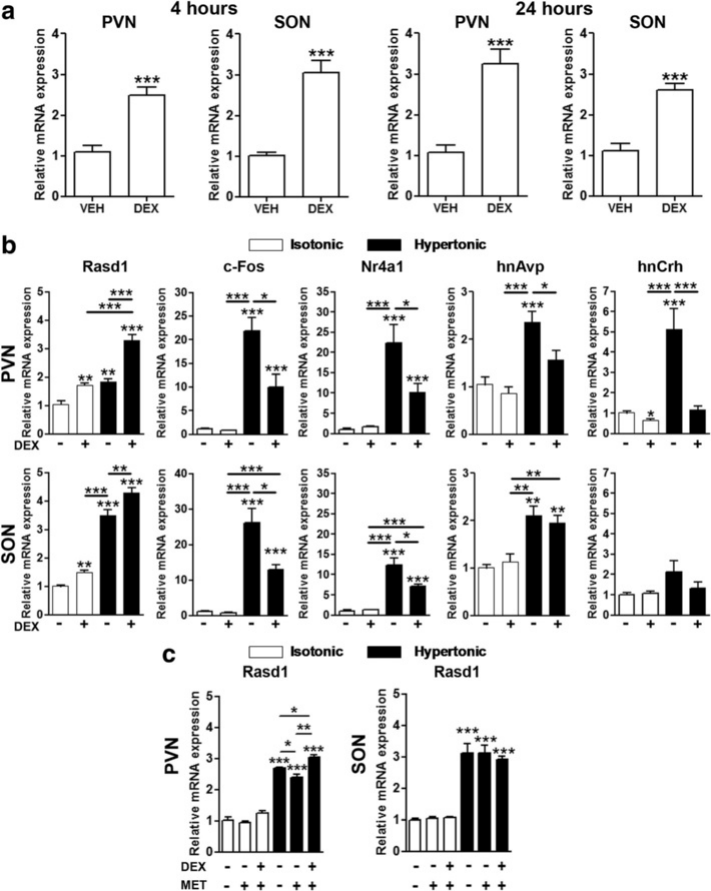
DEX induced *Rasd1* mRNA expression in the rat PVN and SON. **a** the addition of DEX to hypothalamic organotypic culture media 4 or 24 h before collection of samples increased the expression of *Rasd1* in the PVN and SON compared to vehicle (DMSO) controls. **b** rats were injected with DEX (1 mg/kg) or vehicle (0.15 M NaCl) 2 h before i.p injection of IS (0.15 M NaCl) or HS (1.5 M NaCl; 1.5 ml/100 g body weight). Brains were collected 30 min after IS or HS injection. qPCR was performed to analyse the expression of *Rasd1 c-Fos*, *Nr4a1*, *hnAvp* and *hnCrh* in PVN and SON. **c** to block endogenous glucocorticoid synthesis rats were injected with MET or a combination of MET and DEX 4 h before i.p injection of IS or HS. Brains were collected after 30 min and qPCR analysis was performed on the cDNAs generated. **a**, independent-sample unpaired Student’s t tests; **b**–**c**, Two-way ANOVA. Values are means + SEM of *n* = 5−8 animals per group. *p < 0.05, **p < 0.01, ***p < 0.001. DEX, dexamethasone; MET, Metyrapone; VEH, vehicle

Peripheral treatment with DEX has been reported to rapidly and robustly increase the expression of *Rasd1* mRNA in the brain [21]. To determine if *Rasd1* expression was stimulated by glucocorticoids specifically in PVN and SON, we performed ex vivo and in vivo experiments with DEX (Fig. 4). Incubation of hypothalamic organotypic cultures for 4 h and 24 h in the presence of DEX significantly increased *Rasd1* mRNA expression in both the PVN and SON compared to controls (Fig. 4a). Next, to investigate glucocorticoid actions on *Rasd1* expression in vivo, rats were injected with DEX before injection of isotonic (IS) or HS. At 30 min after saline injection mRNA levels were examined in the PVN and SON (Fig. 4b). As expected, HS injection significantly increased *Rasd1* mRNA expression in PVN and SON compared to the vehicle control. DEX injection increased *Rasd1* mRNA expression in PVNs and SONs of rats administered IS, in agreement with our findings for organotypic cultures. However, there was no significant difference in *Rasd1* mRNA in PVNs of rats treated with DEX and IS compared to vehicle and HS, whilst in the SON, HS treatment significantly increased *Rasd1* expression compared to rats treated with DEX and IS. Moreover, DEX injection increased hypertonic induced *Rasd1* mRNA expression in both PVN and SON compared to vehicle controls, perhaps suggesting activation of *Rasd1* expression by separate molecular pathways.

We next focused our attention on the expression of the *Avp* and *Crh* mRNAs, their respective precursor transcripts, heteronuclear *Avp* (*hnAvp*) and heteronuclear *Crh* (*hnCrh*)*,* the assessment of which acts as a surrogate measure for transcription [34, 35]. We also looked at two established cAMP inducible transcription factors, *c-Fos* and nuclear receptor subfamily 4 group A member 1 (*Nr4a1*), the expression of which are known to be induced both by CREB phosphorylation and osmotic stress (reviewed by Yoshida, 2008 [11]). DEX injection reduced HS induced *hnAvp*, *hnCrh, c-Fos* and *Nr4a1* expression in the PVN and *c-Fos* and *Nr4a1* in the SON. Basal levels of gene expression of these genes were unchanged by DEX treatment, with the exception *hnCrh* expression which was lower in the PVN. We then asked if treatment with metyrapone (MET), which blocks corticosterone synthesis [36], could influence basal *Rasd1* mRNA expression, or its response to HS in the PVN and SON (Fig. 4c). Basal *Rasd1* mRNA expression was unchanged by MET treatment in the PVN and SON. However, hypertonic induced *Rasd1* mRNA expression was diminished by pretreatment with MET, though still elevated above control measures, in the PVN and this effect was recovered by combined treatment with DEX. *Rasd1* mRNA expression was unchanged by any of these treatments in the SON.

### DEX and *Rasd1* inhibit cAMP induced gene expression in AtT20 cells

**Fig. 5:**
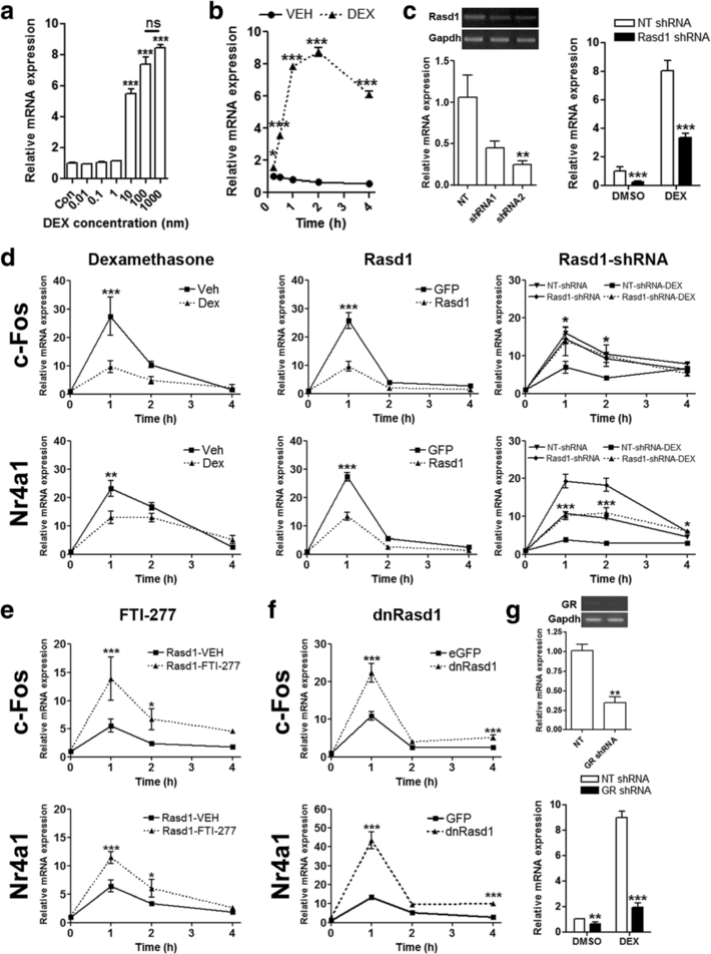
*Rasd1* mediates actions of glucocorticoid on transcription factors *c-Fos* and *Nr4a1* in AtT20 cells. **a**, *Rasd1* mRNA expression in response to 1 h treatment with different concentrations of DEX. **b** changes in *Rasd1* mRNA expression after treatment with 100nM DEX over a 4 h experimental period compared to time matched vehicle controls. **c** responses to *Rasd1* knock down cell lines to 1 h treatment with 100nM DEX were examined by qPCR compared to a control non-targeting shRNA cell line. **d** the effect of pretreatment with DEX (2 h), 72 h of adenoviral mediated *Rasd1* overexpression and *Rasd1* knockdown on FSK (10 μM) induced *c-Fos* and *Nr4a1* mRNA expression was examined compared to controls. **e** cells were infected with *Rasd1* adenovirus for 4 h. Media was replaced and cells were grown in the presence of farensyltransferase inhibitor FTI-277 for 72 h and qPCR analysis of *c-Fos* and *Nr4a1* expression was performed. **f** the effect of 72 h of adenoviral mediated *dnRasd1* overexpression on FSK induced *c-Fos* and *Nr4a1* mRNA expression was examined compared to controls. **g** responses of GR knockdown cell lines to treatment with 100nM DEX were examined by qPCR compared to a control non-targeting shRNA cell line. VEH, vehicle; NT, non-targeting; Con, control. Values are means + SEM of *n* = 3−4 per group. *p < 0.05, **p < 0.01, ***p < 0.001

The stimulatory actions of glucocorticoids on *Rasd1* gene expression were first described in AtT20 cells [18]. We initially treated AtT20 cells with DEX concentrations ranging from 0.01–1000nM to establish a suitable concentration for our studies (Fig. 5a). Maximum stimulation of *Rasd1* mRNA expression was observed at 100nM so we selected this dose for our in vitro studies. Treatment with DEX increased *Rasd1* in a time dependent manner (Fig. 5b), with significance reached at 30 min, and maximum expression between 1–2 h, similar to the effect of acute hypertonic stimulation in vivo (Fig. 1c). We produced shRNAs to knockdown *Rasd1* expression. Of the two *Rasd1* shRNAs we tested, shRNA2 showed significant knockdown of *Rasd1* mRNA (Fig. 5c). We then examined the effect of DEX treatment on *Rasd1* mRNA expression in the *Rasd1* knockdown cell line. The result showed that, from its significantly diminished baseline, *Rasd1* mRNA increased in abundance, suggesting that the knockdown cell line is still able to respond to DEX, but the level of *Rasd1* mRNA expression was significantly reduced compared to the control shRNA cells.

We next asked if the DEX inhibition of *c-Fos* and *Nr4a1* mRNAs seen in vivo was the result of increased *Rasd1* expression. We used FSK to induce endogenous *c-Fos* and *Nr4a1* expression over a time course of 4 h in AtT20 cells pretreated with DEX. As seen in our in vivo study, DEX treatment significantly inhibited *c-Fos* and *Nr4a1* expression in AtT20 cells (Fig. 5d). To establish if *Rasd1* could be mediating these DEX effects, we firstly overexpressed *Rasd1* or eGFP in AtT20 cells using adenoviral vectors, and then treated the transfected cells with FSK to stimulate endogenous *c-Fos* and *Nr4a1* expression. Increasing *Rasd1* expression inhibited FSK stimulation of *c-Fos* and *Nr4a1* with a remarkably similar time course and level of inhibition to that of DEX treatment. shRNA mediated knockdown of endogenous *Rasd1* increased FSK-stimulated *Nr4a1* expression, but not *c-Fos* expression compared to the control shRNA. DEX inhibited FSK-stimulated *Nr4a1* but not c-Fos in both *Rasd1* and non-targeting shRNA cell lines, but expression of *Nr4a1* remained higher than the control cell line. This may be expected as DEX was still able to increase *Rasd1* mRNA in the knockdown cell line (Fig. 5c). When we treated *Rasd1* overexpressing cells with the farnesyltransferase inhibitor FTI-277, a highly potent CAAX peptidomimetic that inhibits Ras signaling [37], FSK-stimulated expression of *c-Fos* and *Nr4a1* increased compared to control (Fig. 5e). Adenoviral mediated overexpression of a CAAX box deficient *Rasd1* (*dnRasd1*) significantly increased *c-Fos* and *Nr4a1* expression confirming the importance of this motif (Fig. 5f).

**Fig. 6:**
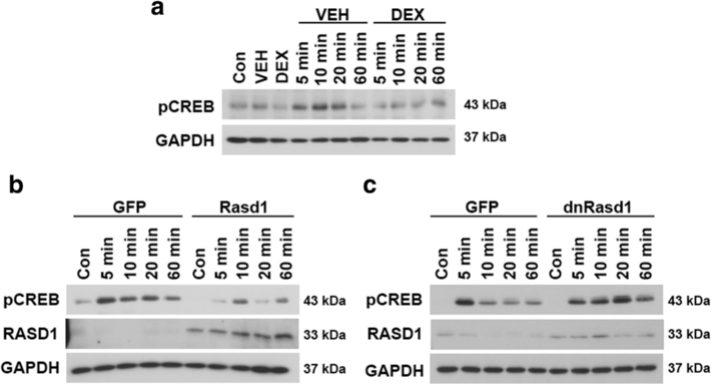
Rasd1 reduces the abundance of phosphorylated CREB in FSK stimulated AtT20 cells. Phosphorylated CREB expression in AtT20 cells (**a**) treated with FSK (5, 10, 20, 60 min) after 2 h pretreatment with DEX, **b** 72 h adenoviral overexpression of *Rasd1* or **c** 72 h adenoviral expression of *dnRasd1* by Western blot. Viral overexpression of Rasd1 was confirmed. GAPDH was used as the loading control. Con, control; VEH vehicle

The GR is believed to be responsible for mediating the effects of DEX on *Rasd1* mRNA expression. Therefore, we produced a GR knockdown AtT20 cell line and tested the responsiveness of *Rasd1* expression to treatment with DEX (Fig. 5g). shRNA mediated silencing of GR expression abolished *Rasd1* stimulation by DEX, confirming that *Rasd1* activation is mediated by GR dependent signaling pathways in AtT20 cells. We speculated that Rasd1 provoked these changes in gene expression may result from altered CREB phosphorylation, which is a known regulator of *c-Fos* and *Nr4a1* (Fig. 6). Indeed CREB phosphorylation was reduced by both DEX (Fig. 6a) and *Rasd1* (Fig. 6b), but was increased by overexpression of *dnRasd1* (Fig. 6c).

### *Rasd1* expression controls the transcriptional response to hyperosmotic stress in vivo

**Fig. 7:**
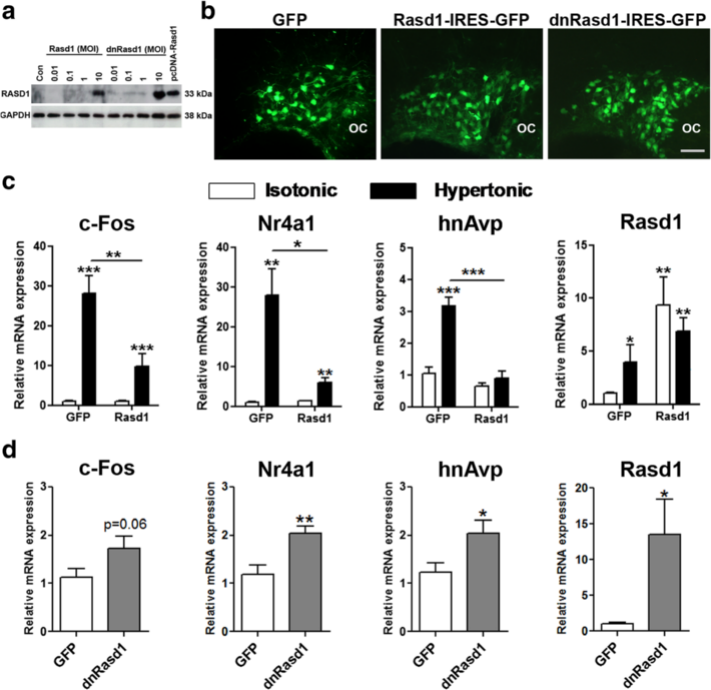
Lentiviral mediated manipulation of *Rasd1* expression in the rat SON alters *c-Fos*, *Nr4a1* and *hnAvp* expression. **a** confirmation of *Rasd1* protein expression 72 h after viral transduction in mouse neuronal cell line N2a. **b** lentiviral transduction of MCN in the SON 2 weeks after injection. **c** rats were injected into SON with lentiviral vectors expressing *Rasd1* or eGFP. Two weeks after viral administration rats received a single i.p injection of either IS (0.15 M NaCl) or HS (1.5 M NaCl; 1.5 ml/100 g body weight). Brains were collected 30 min later. Relative expression of *c-Fos*, *Nr4a1*, *hnAvp* and *Rasd1* was determined by qPCR. **d** two weeks after *dnRasd1* injection rats were presented with 2% (w/v) NaCl solution in place of drinking water for 7 days. Relative expression of *c-Fos*, *Nr4a1*, *hnAvp* and *Rasd1* in SON of *dnRasd1* delivered animals compared to control eGFP control. **c**, Two-way ANOVA; **d**, independent-sample unpaired Student’s t tests. Scale bars = 100 μm. Con, control; MOI, multiplicity of infection. Values are means + SEM of *n* = 4−5 animals per group. *p < 0.05, **p < 0.01, ***p < 0.001

We asked about the transcriptional effects of *Rasd1* overexpression in the SON in vivo. We confirmed expression of the *Rasd1* and *dnRasd1* lentiviruses in N2a cells by immunoblot (Fig. 7a) and in MCN of the SON using the eGFP tag (Fig. 7b). Quantitative PCR confirmed the overexpression of *Rasd1* in the SON (Fig. 7c). We then examined gene expression in SON injected with *Rasd1* compared to eGFP controls. Basal levels of *c-Fos*, *Nr4a1* and *hnAvp* were unaffected by overexpression of *Rasd1* compared to eGFP controls. The expression of all of these genes was increased by HS injection in eGFP virus injected SONs. In *Rasd1* injected SON, HS induced smaller rises in *c-Fos* and *Nr4a1* expression compared to controls and inhibited HS induced *hnAvp* expression. We then directly assessed the importance of *Rasd1* in the hypothalamic systems controlling homeostasis using the SL model in rats injected with *dnRasd1* virus (Fig. 7d). Quantitative PCR confirmed higher expression of *Rasd1* in SON injected with *dnRasd1* compared to eGFP controls. The expression of *dnRasd1* in the SON increased expression of *c-Fos*, *Nr4a1* and *hnAvp* in SL rats compared to eGFP controls. However, the increase of *c-Fos* expression did not reach statistical significance.

## Discussion

*Rasd1* is becoming somewhat of avant-garde member of the Ras family of GTPases by performing many non-conventional signaling functions. Our identification of *Rasd1* in *Avp* neurons starts a new chapter for this small GTPase. Here we show that *Rasd1* is rapidly induced by stress in the PVN, and by elevated plasma osmolality in the PVN and SON of the hypothalamus. We propose that the abundance of RASD1 in MCN and PCNs, based on its inhibitory actions on CREB phosphorylation, is an important mechanism for controlling the transcriptional responses to stressors in both the PVN and SON. In MCN we show, by virally mediated overexpression of *Rasd1*, that *Rasd1* inhibits HS induced stimulation of cAMP inducible genes. When a CAAX box deficient mutant form of *Rasd1* is expressed in the SON cAMP inducible genes were further increased by SL. These effects likely occur through modulation of cAMP-PKA-CREB signaling pathway.

Our interest in *Rasd1* began following identification increased expression of this gene in the SON of the 3 days DH rat using microarrays [28]. Here, using qPCR, we confirmed increased *Rasd1* expression in rat PVN and SON by DH, SL and HS injection. These protocols have all been shown to increase plasma corticosterone levels in the rat [38–41], suggesting that glucocorticoids may be responsible for increased *Rasd1* mRNA expression in the brain. To test this notion, we performed restraint stress, where plasma corticosterone levels are increased [42, 43] in the absence of the osmotic cues presented by hyperosmotic stress. To our surprise, restraint stress rapidly increased *Rasd1* expression, but only in the PVN, suggesting differing mechanisms of *Rasd1* activation in the PVN and SON by restraint stress and osmotic stress. We show here in AtT20 cells that the GR is important for *Rasd1* induction by DEX similar to previous reports for other cell lines [44]. We thus proposed that the location of GRs in the hypothalamus likely underlies these differing responses to restraint stress. Indeed, in support of this concept, PCNs of the PVN are known to express comparatively higher levels of the GR than MCN of the SON [14–17]. These data thus imply different mechanisms of *Rasd1* activation in PCNs and MCN.

The observed expression of *Rasd1* exclusively in AVP positive neurons suggested a role in the regulation of these neurons. Such regulation may occur at multiple levels: as we observed *Rasd1* expression in neuronal cell body, dendrites and axons of control rats. The presence of *Rasd1* in axons is novel, but this property is not unique to this particular Ras protein. In the mouse hippocampus another member of the Ras family, H-Ras, a potent activator of ERK signaling, has previously been identified in axon terminals [45]. Transgenic mice overexpressing a constitutively active mutant of H-Ras, which strongly localises to axon terminals in the hippocampus, resulted in several presynaptic changes including a higher density of docked neurotransmitter vesicles in glutamatergic terminals [45]. The granular cytoplasmic pattern of RASD1 immunoreactivity in the neuronal cell body suggests that RASD1 may be associated with vesicular structures in the cytoplasm. The presence of RASD1 in cell processes suggests that RASD1 has secretory role in AVP neurons. The idea that *Rasd1* could influence hormone secretion is not new. A number of in vitro studies have reported inhibitory actions of *Rasd1* on hormone secretion but the precise mechanisms mediating these inhibitory effects remains to be established [21, 46–48]. It is established that high plasma osmolality results in the secretion and depletion of AVP in MCN cell bodies, even though mRNA expression of *Avp* is up-regulated in the PVN and SON [49, 50]. This is also true for RASD1 in SL rats. What is known is that RASD1 regulates a number of cell signaling processes [51, 52], in particular it has inhibitory actions on the cAMP-PKA-CREB signaling pathway [21–25]. Therefore, any changes in cellular abundance of RASD1, as we observe here in the MCN cell bodies by hyperosmotic stress and the increase in PCN by restraint stress, would be expected to influence the cAMP signalling pathway.

The expression of *Rasd1* in the brain has been shown to be under hormonal control [20]. We show here that DEX increases *Rasd1* expression in the PVN and SON of the hypothalamus. These findings differed from our observations in restraint stress where *Rasd1* increased only in the PVN. One possibility for these differences is the greater potency of DEX in its glucocorticoid effects than endogenous corticosterone. Our data showed that pretreatment with DEX potentiated HS induced increases in *Rasd1* expression in PVN and SON, consistent with our hypothesis of two separate mechanisms activating *Rasd1* transcription in these nuclei. This was confirmed by treatment with MET, which decreased *Rasd1* expression only in the PVN, a response that was effectively rescued by DEX. These findings are consistent with the presence of GRs in MCN of the SON a well as PCN of the PVN [15, 16]. These data thus imply two separate mechanisms of *Rasd1* activation in the PVN and SON, one sensitive to glucocorticoids, and one mediated by a currently undefined mechanism in response to elevated plasma osmolality.

The negative feedback of glucocorticoids, in our case using DEX, on *hnAvp* and *hnCrh* transcription in PVN has been known for many years [7, 53–55]. Withdrawal of glucocorticoids by adrenalectomy results in increased *Crh* and *Avp* mRNA expression in the PVN [56, 57], which can be reversed by glucocorticoid replacement [7]. In adrenalectomised rats, by replacing corticosterone, Kovacs et al. demonstrated that glucocorticoids selectively targets *Avp* transcription in PCNs and not MCN of the PVN [7]. In PCNs of the rat PVN, these transcriptional events are thought to be mediated by stress induced increases in CREB phosphorylation, which results in increased *Crh* and *Avp* transcription [8, 9, 12]. Inhibition CREB phosphorylation is thought to be one of the targets for the negative feedback of glucocorticoids on *Avp* and *Crh* transcription in PCN of the PVN [7, 12]. Furthermore, it is known that cAMP increases in the SON in response to hyperosmotic stress [58–60], through activation of the PKA pathway and subsequent CREB phosphorylation [13], and this is thought to stimulate *Avp* transcription.

In recent years many in vivo actions of DEX have been attributed to molecular signaling through *Rasd1* [44, 46, 47] suggesting that DEX actions in the hypothalamus were the result of increased *Rasd1* expression. Our in vivo observations of altered *c-Fos* and *Nr4a1* expression by DEX treatment in the PVN and SON, where *Rasd1* expression is elevated, prompted us to compare DEX treatment with *Rasd1* overexpression on gene expression in mouse corticotroph AtT20 cells. Both *c-Fos* and *Nr4a1* are well established cAMP inducible genes that are induced by phosphorylated CREB [61], and respond robustly to increases in neuronal activity arising from osmotic stress and restraint stress [62, 63]. There is also evidence that DEX inhibits *c-Fos* and *Nr4a1* expression [53, 55, 64], so we speculated that these effects could be mediated by *Rasd1* via modulation of cAMP-PKA-CREB signaling pathway. We show by overexpression and knockdown, that *Rasd1* can reproduce DEX effects on *c-Fos* and *Nr4a1* in AtT20 cells, suggesting that glucocorticoids primarily act through *Rasd1* to inhibit the expression of these transcription factors.

RASD1 has also been shown to inhibit cAMP inducible genes through interactions at the promoter. RASD1 interacts with NonO, a member of RNA-Recognition motif gene family, at cAMP response element (CRE) sites within target genes, including *Nr4a1*, inhibiting their transcription [27]. Furthermore, in cortical neuron cultures, transfection of a CRE driven luciferase construct also demonstrated the inhibitory actions of RASD1 on FSK induced CRE mediated transcription [26]. The transcriptional changes in the present study were isoprenylation dependent as determined using the farnesyltransferase inhibitor FTI-277 and the CAAX deficient *dnRasd1* suggesting membrane translocation is necessary. RASD1 undergoes posttranslational modifications by farnesylation of its CAAX box [25] similar to other members of the *Ras* family [65]. Isoprenylation of this CAAX consensus site is required for translocation of *Ras* proteins to the cell membrane [65, 66], stimulation of protein-protein interactions, and can affect protein stability [67]. Isoprenylation has been shown to reduce the stability of *Ras* proteins [68] and is thought to be necessary for rapid protein turnover of GTPase RhoB [69]. The decreased stability of farnesylated proteins may provide an explanation for the lower levels RASD1 observed in the PVN and SON during SL, which may be the result of increased turnover of RASD1 protein. These data imply that the observed actions of *Rasd1* in AtT20 cells occur through regulation of G proteins at the plasma membrane.

The expression of over 100 non-chemosensory G protein coupled receptors has been described in the PVN and SON [70], many of which could be subject to regulation by RASD1. Indeed, it has been proposed that RASD1 may act to antagonise G protein coupled receptor signaling by altering the pool of heterotrimeric G-proteins available for receptor coupling [71]. Interestingly, *Rasd1* has been reported to selectively activate transient receptor potential channel 4 (TRPC4) in INS-I cells [72], and altered expression of these channels has been described in vasopressinergic MCNs of the PVN and SON in response to hyperosmotic stress [28, 73]. The activation of TRPC4 in neurosecretory cells is known to trigger a robust secretory response [74], suggesting that *Rasd1* may affect the secretion of vasopressin. Furthermore, *Rasd1* has been shown to modulate N-type calcium channels in HEK293 cells [75]. Thus, *Rasd1* has the capacity to modulate calcium influx into cells and may influence calcium dependent events such as vasopressin secretion from MCNs. Therefore, in hypertonic stress altered RASD1 expression may perhaps modulate the excitability of MCNs by altering channel activity, though this remains to be determined.

*Rasd1* overexpression has been shown to inhibit GαS and FSK mediated activation of AC through activation of Gαi and Gβγ [22]. Interestingly, SL in rats increases Gαi expression in MCN of the PVN and SON [60] consistent with the idea of inhibitory inputs on cAMP production in osmotic stress. In addition, *Rasd1* has been seen to abolish dopamine D2L receptor mediated potentiation of AC2 activity by blocking protein kinase C and Gβγ activity [25], and to block dopamine D2L receptor mediated heterologous sensitization of AC1 [24]. Both AC1 and AC2 have been reported in the PVN and SON [76], so could be targets of *Rasd1* actions in these brain nuclei as well as other brain regions that express *Rasd1*.

To validate our in vitro findings in the physiological context of the whole organisms, we used lentiviral vectors to overexpress *Rasd1* in the rat SON. Analogous to our findings in AtT20 cells, overexpression of *Rasd1* in the SON reduced HS induced increases in *c-Fos* and *Nr4a1*, and completely blocked induced *hnAvp* expression. Therefore, *Rasd1* alone could mimic the actions of DEX on these target genes in the SON, suggesting that DEX effects in PVN and SON are mediated through altered *Rasd1* expression. We suggest that this is due to inhibition of CREB phosphorylation. In a recent in vivo study, injection of a recombinant adeno-associated virus expressing ACREB, a dominant negative inhibitor of endogenous CREB, into the SON resulted in decreased *c-Fos* and *Nr4a1* mRNA expression [77]. Furthermore in the SCNs of *Rasd1* knockout mice, Cheng et al. [26] reported higher cAMP levels, alongside increased *c-Fos* and CREB phosphorylation, compared to wild-type mice. These changes in *Rasd1* knockout mice are consistent with removal of the inhibitory influences of *Rasd1* on cAMP dependent signaling pathways. This is consistent with our findings with *dnRasd1* overexpression in the SON of the SL rat where expression levels of cAMP inducible genes were increased. We would predict a similar mode of action for *Rasd1* overexpression in PCNs of the PVN based on our findings presented here. Unfortunately, our viruses are not able to selectively discriminate between MCN and PCNs of the PVN to extend these findings to PCNs of the PVN.

RASD1 has been shown to exist in ternary complex with carboxy-terminal PDZ ligand CAPON and neuronal nitric oxide synthase (nNOS) [30]. This enables activation of *Rasd1* by s-nitrosylation on single cysteine (Cys11) residue at the N-terminus of this protein [78]. In cortical neuronal cultures, *Rasd1* is activated by nitric oxide donors as well as N-methyl-D-aspartate receptor stimulated nitric oxide synthesis [78]. The AVP expressing MCN of the SON express nNOS, RASD1, CAPON [30] and *nNos* is also subjected to regulation by DH and SL [79, 80]. We do not know whether RASD1 complexes with nNOS and CAPON regulate any signaling events in the SON, or indeed if s-nitrosylation of RASD1 is important. However, we do know that AtT20 cells do not express *nNos* [81], so *nNos* is not a necessary component for *Rasd1* mediated inhibition of cAMP pathways.

## Conclusion

**Fig. 8:**
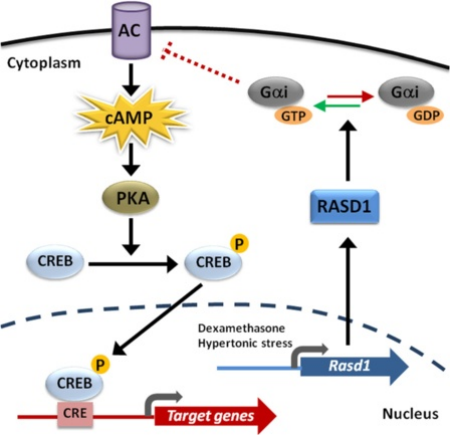
Proposed mechanism of action of *Rasd1* in cAMP-dependent gene regulation. *Rasd1* mRNA expression is upregulated by glucocorticoid (dexamethasone) and increased plasma osmolality (hypertonic stress). In the cytoplasm RASD1 undergoes posttranslational processing by isoprenylation of its CAAX box motif enabling trafficking to the cell membrane. RASD1 activates Gαi which inhibits the cAMP dependent pathway by inhibiting adenylyl cyclase. This results in the inhibition of the cAMP-PKA-CREB signalling pathway

In summary, our results showed that *Rasd1* is expressed in vasopressinergic neurons in the PVN and SON of the hypothalamus. We identified two mechanisms of *Rasd1* induction in the hypothalamus, one by elevated glucocorticoids in response to stress, and one in response to increased plasma osmolality resulting from osmotic stress. The ability to influence *c-Fos*, *Nr4a1*, and *hnAVP* in vivo by lentiviral gene transfer greatly strengthened our hypothesis that *Rasd1* was inhibiting cAMP-PKA-CREB signaling pathway in the hypothalamus (Fig. 8). We propose that the abundance of RASD1 in MCN, based on these inhibitory actions, is important for controlling the transcriptional response to osmotic stress in the PVN and SON. In PCNs of the PVN, *Rasd1* has all the necessary credentials to facilitate the rapid glucocorticoid negative feedback of the HPA axis on *Crh* and *Avp* expression. Studies have long proposed that an intermediate signalling molecule activated by the GR may be necessary for glucocorticoid negative feedback on CREB phosphorylation and our data points towards this being *Rasd1*.

## Methods

### Animals

Male Sprague–Dawley rats weighing 250–300 g were used in this study, except for the restraint stress experiment (performed in Brazil), where male Wistar rats weighing 250–300 g were used. Rats were maintained under a 14:10 light dark cycle (lights on at 0500) with food and water ad libitum for at least 1 week prior to experimentation. Animal experiments were performed between 9 am–2 pm. All experiments in the UK were performed under a Home Office UK licence held under, and in strict accordance with, the provision of the UK Animals (Scientific Procedures) Act (1986); they had also been approved by the University of Bristol Animal Welfare and Ethical Review Board. The experiments in Brazil were conducted according to the “Guide for the Care and Use of Laboratory Animals” (NIH Publication No. 85–23, revised 1996), and experimental protocols were approved by the local Ethics Committee on Animal Use in the School of Medicine of Ribeirão Preto, University of São Paulo.

### Hypertonic stress experiment

We used two protocols a chronic and an acute hypertonic stress protocol. To induce chronic hyperosmotic stress, water was removed (DH) for 1 or 3 days or replaced by 2% (w/v) NaCl in drinking water for 1 or 7 days SL. The control group had access to food and water ad libitum. In some instances water was returned after DH and SL for 24 h (rehydration). The acute responses were assessed (10 min, 30 min, 1, 2, or 4 h) after a single i.p injection of 1.5 ml/100 g body weight of 1.5 M NaCl solution HS. After injection, HS rats were placed back in their home cages and water was removed. The reference group (time 0) had access to food and water ad libitum. In acute experiments with time-point comparisons the control groups received a single i.p injection of IS solution (1.5 ml/100 g body weight of 0.15 M NaCl solution).

### Restraint stress experiment

Rats were randomly allocated to 3 groups (control, 0.5 h and 1 h restraint). The control group stayed in their home cages and had access food and water ad libitum throughout the experimental period. For RNA analysis, animals were placed in clear plexiglass tubes with access to air for either 0.5 h or 1 h duration, before being immediately killed. For immunofluorescence protein analysis, the rats were killed 3 h after removal from the tube to allow time for *de novo* protein synthesis to take place.

### Glucocorticoid studies

To investigate actions of glucocorticoid on gene expression in the hypothalamus, rats were injected intraperitoneally with 0.5 ml 0.15 M NaCl (vehicle) or 0.5 ml of 1 mg/kg body weight DEX (Sigma D2915) 2 h before IS or HS injection. To inhibit the synthesis of endogenous glucocorticoid, rats were injected subcutaneously with 0.5 ml sesame oil (vehicle), 150 mg/kg body weight MET (Sigma 856525), which inhibits endogenous corticosterone synthesis by inhibiting steroid 11-β hydroxylase activity, or 150 mg/kg MET and 0.2 mg/kg body weight DEX 4 h before IS or HS injection. After injection of HS or IS rats were placed back in their home cages, and water, but not food, was removed for the remaining 30 min of the experiment. For RNA analyses, rats were killed by striking of the cranium, followed by decapitation, using a guillotine (Harvard Apparatus). Brains were removed and frozen on dry ice before being stored at −80 °C.

### RNA extraction and cDNA synthesis

Frozen brains were sliced into 60 μm coronal sections in a cryostat. Sections were mounted on glass slides and stained with 0.1% (w/v) toludine blue then visualised on a light microscope until brain nuclei were visible, then SON and PVN samples were collected using a 1 mm micro punch (Fine Scientific Tools). The optic chiasm (SON), or neurons lateral to the third ventricle (PVN), were used as a reference. SON and PVN samples were then dispensed into 1.5 ml tubes and kept on dry ice within the cryostat. Total RNA was extracted from punched samples by combining Qiazol Reagent with Qiagens RNeasy kit protocols (Qiagen). The punched samples were removed from dry ice and rapidly resuspended, by vortexing, in 1 ml Qiazol reagent. Following Qiazol phase separation with chloroform, 350 μl of the upper aqueous phase was removed, mixed with 350 μl 70% (v/v) ethanol and applied to RNeasy columns. The remaining steps were performed as recommended by the manufacturer. For cDNA synthesis 200 ng (tissue) or 500 ng (cells) of total RNA was reverse transcribed using the Quantitect reverse transcription kit (Qiagen).

### Real-time quantitative PCR analysis

Primers for *Rasd1* (Rat/Mouse, 5’-CCCTCAGCGTTGTGCCTACT-3’ and 5’-AAAGAGCGCACGGAACATCT-3’), *c-Fos* (Rat, 5’-AGCATGGGCTCCCCTGTCA-3’ and 5'-GAGACCAGAGTGGGCTGCA-3'; Mouse, 5’-TCCCCAAACTTCGACCATGA-3’ and 5’-GGCTGGGGAATGGTAGTAGG-3’), *Nr4a1* (Rat, 5'-CTGCGACTGGGTCCTGGGTC-3' and 5'-TGTCAGGTGGTCACGCGGTC-3'; Mouse, 5’-AAAGTTGGGGGAGTGTGCTA-3’ and 5’-GAATACAGGGCATCTCCAGC-3’), rat *hnCrh* (5'-GGGCGAATAGCTTAAACCTG-3' and 5'-CAGGTGACCCTTCCTTGGAGA-3'), mouse GR (5’-TGTCACTGCTGGAGGTGATT-3’ and 5'-ATCACTTGACGCCCACCTAA-3'), rat *hnAvp* (5'-GAGGCAAGAGGGCCACATC-3' and 5'-CTCTCCTAGCCCATGACCCTT-3'), rat mature *Avp* (5'-TGCCTGCTACTTCCAGAACTGC-3' and 5'-AGGGGAGACACTGTCTCAGCTC-3'), eGFP (5’-ATCATGGCCGACAAGCAGAAGAAC-3’ and 5’-GTACAGCTCGTCCATGCCGAGAGT-3’), rat *Rpl19* (5'-GCGTCTGCAGCCATGAGTA-3' and 5'-TGGCATTGGCGATTTCGTTG-3') and *Gapdh* (Rat, 5’-ATGATTCTACCCACGGCAAG-3’ and 5’-CTGGAAGATGGTGATGGGTT-3’; Mouse, 5’-CAACTCCCACTCTTCCACCT-3’ and 5’-CTTGCTCAGTGTCCTTGCTG-3’) were synthesised by Eurofins MWG Operon. The qPCRs were carried out in duplicate using SYBR green (Roche) on an ABI StepOnePlus Sequence Detection System (ABI, Warrington, UK). For relative quantification of gene expression the 2^-ΔΔCT^ method was employed [82]. The internal control gene used for these analyses were the housekeeping gene *Rpl19* and *Gapdh*. To analyse *Rasd1* and GR gene knockdown PCRs for gel electrophoresis were performed using *Taq*DNA polymerase (New England Biolabs).

### Immunofluorescence

Rats were deeply anesthetised with sodium pentobarbitone (100 mg/kg i.p.) and transcardially perfused with 0.1 M phosphate buffered saline (PBS, pH 7.4) followed by 4% (w/v) paraformaldehyde (PFA) in PBS. Brains were removed and post-fixed overnight in 4% (w/v) PFA followed by 30% (w/v) sucrose prepared in PBS. Tissues were sectioned to 40 μm on a cryostat, washed in PBS and blocked for 30 min in 5% (v/v) horse serum in PBS containing 0.25% (v/v) Triton X-100 (PBST). Sections were incubated with 1:500 dilution of rabbit anti-RASD1 antibody (Abcam, ab78459), 1:100 mouse anti-OT (neurophysin-I, PS38, [83]) or 1:100 mouse anti-AVP (neurophysin-II, PS41) prepared in 1% (*v*/*v*) horse serum in PBST at 4 °C overnight. The Rasd1 antibody has previously been shown to detect Rasd1 in mouse brain extracts by Western blot (data sheet), mouse kidney by immunohistochemistry (data sheet), and rat pancreas by immunofluorescence [47]. The sections were washed three times in PBS for 5 min and incubated with 1:500 dilution of anti-rabbit IgG-biotinylated secondary antibody in PBST for 1 h at room temperature. Sections were washed three times for 5 min with PBS and incubated for 1 h with secondary antibodies conjugated with fluorophore Alexa Fluor 488 streptavidin-conjugated and Alexa Fluor 594 donkey anti-mouse (Invitrogen). Sections were mounted and sealed with VectorShields mounting media (Vector Laboratories).

The GFP-Rasd1 fusion construct was generated by overlap extension PCR. Primers for GFP (5’-CGCGGATCCATGGTGAGCAAGGGCGAGGA-3’ and 5’-CTTGTACAGCTCGTCCATGCCGA-3’) and Rasd1 (5’-TCGGCATGGACGAGCTGTACAAGAAACTGGCCGCGATGAA-3’ and 5’-ACGCGTCGACCTAACTGATGACACAGCGCT-3’) were used for PCRs using Phusion High-FidelityDNAPolymerase (New England BioLabs). The final PCR product was digested with BamH1 and SalI and cloned into the corresponding sites of pcDNA3.1. N2a cells were grown on coverslips in 12-well tissue culture plates and transfected with GFP-Rasd1 fusion construct using Lipofectamine LTX (Life Technologies). At 48 h after transfections cells were fixed with 4% (*w*/*v*) PFA in PBS for 10 min and washed three times with PBS for 5 min. Cells were then incubated with 0.3% (*v*/*v*) Triton X-100 in PBS for 10 min for permeabilisation followed by 5% (v/v) horse serum prepared in PBS with 0.03% (*v*/*v*) Triton X-100 for 30 min for blocking. Cells were incubated with 1:500 dilution of rabbit anti-RASD1 antibody (Abcam, ab78459) prepared in 1% (*v*/*v*) horse serum in PBS-0.03 T at 4 °C overnight. After three washes, cells were incubated with Alexa Fluor 594 donkey anti-rabbit IgG (1:500; Invitrogen) for 1 h at room temperature, followed by three washes with PBS-0.03 T. Coverslips were mounted onto glass slides using VectorShields hard mounting media with DAPI. Images were captured on a confocal microscope (Leica).

### Protein extraction and immunoblotting

Cells were washed twice with cold PBS pH 7.4 (Gibco; 10010–015), and harvested by scraping into RIPA buffer, consisting of PBS containing 1% (*v*/*v*) IGEPAL CA-630 (Sigma I3021), 0.5% (*w*/*v*) sodium deoxycholate, and 0.1% (*w*/*v*) sodium dodecyl sulfate, 1 mM PMSF, protease inhibitor (Sigma: P8340) and phosphatase inhibitor (Roche: 04906845001). The lysate was incubated on ice for 15 min with vortexing every 5 min, followed by centrifugation at 10,000 × g for 10 min. Supernatants were collected and kept at −80 °C. Protein concentrations were determined using the Bradford assay (Bio-Rad).

For immunoblotting, proteins (50 μg) were separated by SDS-PAGE and transferred to 0.45 μm PVDF membranes (Millipore). The membranes were blocked with 3% (*w*/*v*) ECL Prime blocking agent (GE Healthcare) in Tris-buffered saline-Tween 20 (0.1% (*v*/*v*) TBS-T) for 1 h at room temperature, followed by incubation with primary antibody diluted in 3% (*w*/*v*) ECL Prime blocking agent in TBST at 4 °C overnight. This was followed by incubation with appropriate secondary antibody conjugated with horseradish peroxidase (HRP) at room temperature for 1 h. Membranes were washed with TBST. Signal was visualized using high sensitivity WESTAR EtaC or WESTAR SuperNova extreme sensitivity HRP Detection Substrate (Cyanagen). Primary antibodies used: a rabbit polyclonal anti-RASD1 (1:1000; Abcam, ab78459), goat polyclonal anti-phosphoCREB (1:2500; Santa Cruz, sc-7978), and a mouse polyclonal anti-GAPDH (1:20,000; Santa Cruz, sc-32233) antibody. Immunoblots were stripped in Restore Western blot stripping buffer (ThermoScientific) and re-probed to assess multiple proteins in the same blot.

### Organotypic studies

Organotypic cultures were prepared as described previously [84]. Sprague–Dawley pups (P5–P7) were purchased from Harlan Laboratories (UK). Pups were decapitated using scissors, and brains were removed and incubated in cold-Hank’s solution for 5 min, and then dissected using the optic chiasm as a landmark to produce a hypothalamic block. Slices (400 μm) were cut on a Mcllwain Tissue Chopper and placed onto hydrated Millipore Millicell-CM filter inserts in 6 well tissue culture plates containing 1.1 ml of culture medium. After 10 day, culture medium was replaced with serum free medium. The cultures were incubated at 35 °C in 5% (v/v) CO_2_ enriched air and medium was replaced every 2 days. Experimental treatments were performed after 4 days in serum free medium. Slice cultures were incubated with 0.01% (*v*/*v*) DMSO (vehicle) or 100nM DEX prepared in serum free medium for 4 h or 24 h. The inserts were frozen on dry ice in 6-well tissue culture plates, SON and PVN were punched (1 mm diameter micropunch) from 3 slices in a cryostat and RNA was extracted as described above.

### Cells and treatments

Mouse pituitary cell line AtT20/D16v-F2 (Sigma; 94050406), Human Embryonic Kidney cells HEK293T/17 (ATTC CRL-11268), and Mouse Neuroblastoma N2a cells (ATTC CCL-131) were cultured in DMEM (Sigma; D6546) supplemented with 10% (*v*/*v*) heat-inactivated fetal bovine serum (Gibco), 2 mM L-glutamine and 100 unit/ml of penicillin-streptomycin. Cells were incubated at 37 °C in a humidified incubator with 5% (*v*/*v*) CO_2_. For chemical treatments, cells were seeded onto tissue culture plates to 60–70% confluence. After 24–72 h chemical treatments were performed at the time points indicated in the figure legend; 10 μM FSK (Sigma: F6886) and/or 100nM DEX (Sigma: D1756), or 10 μM FTI-277 (Sigma). Stock solutions of FSK (10 mM) and DEX (100 μM) were prepared in DMSO while FTI-277 (2.5 mM) was prepared in water.

To produce knockdown cell lines, AtT20 cells were transduced with a lentivirus containing shRNAs targeting mouse *Rasd1* or GR. The shRNA sequences (*Rasd1* shRNA1-GCCGTTTCGAGGATGCTTCAA, shRNA2-GCTCAAACAGCAGATCCTAGA; GR- GGAGATACAATCTTATCAAGC) were obtained from the RNAi consortium shRNA library. Sense and antisense oligonucleotides for shRNAs were synthesised (European MWG Operon) and cloned into lentiviral transfer vector pLKO.1 puro according to manufacturer’s guidelines (pLKO.1 puro was a gift from Bob Weinberg, Addgene plasmid 8453) [85]. A non-targeting shRNA sequence (ATCATGTTAGGCGTACGGACT) was used as a control. Virus particles were produced as previously described [86]. Twenty four hours after transduction, culture media was replaced with fresh media containing puromycin (2 μg/ml, Life Technologies). The cells were cultured in presence of puromycin for two weeks before use in experiments. The level of knockdown was confirmed by qPCR.

### Adenoviral vector synthesis

cDNA clones encoding mouse *Rasd1* and a *dnRasd1* were the kind gift of Professor Richard Dorin, University of New Mexico. cDNAs were excised from pcDNA3.1 with restriction enzymes KpnI and XhoI and ligated into compatible restriction sites of adenoviral vector pacAd5.CMV (Cell Biolabs). Adenoviral vector pacAd5.CMV.eGFP was used as a control. The adenoviruses were generated by co-transfection of viral shuttle and backbone (pacAd5 9.2-100) vectors in HEK293T cells by calcium phosphate method in accordance with manufacturer’s guidelines (Cell Biolabs). Adenoviruses were purified by two rounds of CsCl ultracentrifugation and desalted using Slide-A-Lyzer dialysis cassettes (Thermoscientific). The purified viruses were aliquoted and stored at −80 °C. The virus titers were determined in triplicate by standard plaque assay. A multiplicity of infection of 10 was used for cell experiments.

### Lentiviral vector gene transfer into SON

We used lentiviral vectors to study *Rasd1* overexpression in the rat brain due to their higher infectivity of neurons compared to adenoviruses [87]. The cDNA clones of *Rasd1* and *dnRasd1* were excised from plasmid pcDNA3.1 and expressed in lentiviral vector pRRL.SIN.CPPT.CMV.IRES.eGFP.WPRE (modified from Addgene plasmid 12252). A lentiviral vector expressing eGFP (pRRL.SIN.CPPT.CMV.eGFP.WPRE) was used as a control. Viruses were generated as described previously [86]. Titers were calculated in transduction units per ml (TU/ml) with all viruses in the present study having a titer of > 5 x 10^9^ TU/ml.

Stereotaxic injections of lentiviral vectors into the SON were performed as previously described [84]. Lentiviral vector tropism was assessed by visualising GFP expression in perfused tissue. To investigate gene expression following overexpression of *Rasd1* or *dnRasd1* in the SON, rats received bilateral lentiviral injections, where *Rasd1* or *dnRasd1* virus was administered to one nuclei and eGFP into the other and viral expression was allowed to proceed for two weeks. Two weeks after *Rasd1* virus administration rats received a single i.p injection of either IS (0.15 M NaCl) or HS (1.5 M NaCl; 1.5 ml/100 g body weight) and were killed 30 min later. Two weeks after *dnRasd1* virus administration rats were SL for 7 days.

### Statistical analysis

Statistical differences between two experimental groups were evaluated using independent-sample unpaired Student’s t tests. One-way ANOVA with Tukey’s post hoc test were used to determine the difference between more than two samples with only a single influencing factor. Two-way ANOVA with Bonferonni post hoc test was used to determine interactions between two independent variables on the dependent variable. *p* < 0.05 was considered significant.
